# Large-scale brain dynamics: effect of connectivity resolution

**DOI:** 10.1186/1471-2202-16-S1-O20

**Published:** 2015-12-18

**Authors:** Timothée Proix, Andreas Spiegler, Viktor K Jirsa

**Affiliations:** 1 Aix Marseille Université, Inserm, INS UMR_S 1106, 13005, Marseille, France

## 

Large-scale brain dynamics recently started to be modeled numerically based on both heterogeneous large-scale networks build from diffusion MRI, that is, a connectome, and local homogeneous connectivity kernel representing intracortical synaptic connections. However, topological properties of a connectome can significantly change with resolution and parcellation [1]. Furthermore, the sampling of the cerebral surfaces, resulting in a geometric model, and, in this way, the local connectivity kernel play crucial roles in the formation of spatial patterns on the cerebral surfaces as well as on the sensor level (e.g., EEG electrodes) by a forward calculation [2]. However, the effect of sampling and parcellation on modeling brain dynamics has not been studied so far. Here, we investigate qualitatively and quantitatively: (i) how different parcellation resolutions affect the dynamics of the network; and (ii) how the local connectivity affects the network dynamics. To do so, we used the neuroinformatics platform for large-scale brain simulations, called The Virtual Brain (TVB) [3] and developed a preprocessing pipeline to incorporate experimental data (e.g., structural MRI, diffusion-weighted MRI) in TVB [4].

We prepared ten individual models based on ten randomly selected subjects from the Human Connectome Project dataset [5]. For each individual model we performed simulations under two conditions during rest: (i) noise driven, using a bistable neural mass model, and (ii) after stimulation, using an excitable neural mass model. We investigated the effect of heterogeneous and homogeneous connectivity on large-scale brain dynamics by different numbers of regions in the parcellation (70 to 2240) and by varying the local connectivity coupling strength. To introduce experimental data (i.e., structural and diffusion MRI) into TVB we tackled issues such as surface downsampling (for achieving moderate simulation times) and mapping between surface and parcellation (to consistently use heterogeneous and homogeneous connectivity) by developing the Surface and Connectivity Reconstruction with an Imaging Pipeline for TVB Simulations, short SCRIPTS [4].

When considering slow dynamics, the major fiber bundles best reflected in the coarsest parcellation appeared to be mainly responsible for the emergence of the network attractors with limited changes over different parcellations and different local coupling strengths. For fast dynamics, new qualitative solutions appeared, but only in the presence of delays.

**Figure 1:**
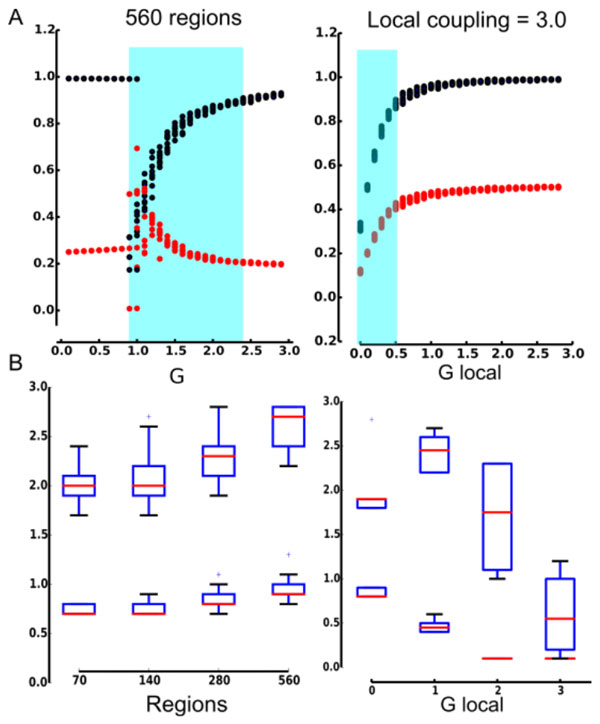
**A. Spatial attractors. For each value of the global (G) or local (G_local) coupling parameter, correlation with in-strength (blue points) and with s-core (red points) for ten different initial conditions**. The blue square indicates the critical interval. B. Values for the beginning and end of the critical range as a function of G or G_local.
